# Supplementary material for: Evaluation of Genistein as a Mitochondrial Modulator and Its Effects on Sperm Quality
Source: Int J Mol Sci. 2023 Sep 19;24(18):14260. doi: 10.3390/ijms241814260 (PMC10531583; doi:10.3390/ijms241814260)

Supplementary Figure S1: Sum of testicles weight. Results are presented as mean ± standard deviation from the sum of right and left testicles (n = 8 animals per group). Control group: 3.67 ± 0.21 g. Genistein treated group: 3.44 ± 0.18 g. Statistical analysis was performed with Mann-Whitney test (* $p < 0.05$).

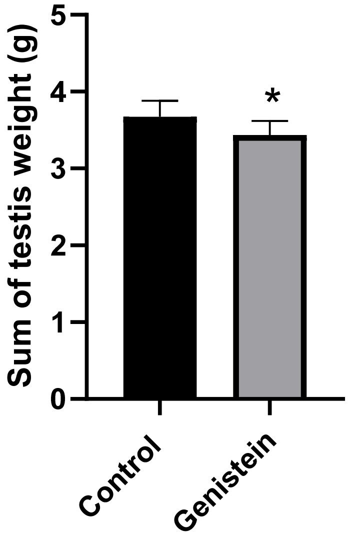

Supplement: Supplementary file 1 [file ijms-24-14260-s001.zip › ijms-2588462-supplementary.pdf]
